# Effect of TGase Crosslinking on the Structure, Emulsification, and Gelling Properties of Soy Isolate Proteins

**DOI:** 10.3390/foods14122130

**Published:** 2025-06-18

**Authors:** Ziqi Peng, Kunlun Liu, Ning Liao

**Affiliations:** College of Food Science and Engineering, Henan University of Technology, Zhengzhou 450001, China; ppzq2002@163.com (Z.P.); liaoning@haut.edu.cn (N.L.)

**Keywords:** protein nanoparticles, transglutaminase, crosslinking, conformational changes, properties

## Abstract

Soy isolate protein (SPI), as a high-quality plant protein source, is often processed into various soy products. In this study, the physicochemical properties of SPI treated with transglutaminase (TGase) were investigated in correlation with emulsification characteristics and rheological behavior. The polyacrylamide gel electrophoresis with sodium dodecyl sulfate (SDS-PAGE) and Fourier-transform infrared spectroscopy (FTIR) and endogenous fluorescence spectrum analysis results showed that TGase was able to promote the covalent binding of lysine and glutamine residues in SPI. The moderate pre-crosslinking treatment of TGase (5–7.5 U/g TGase pre-crosslinked for 2 h or 5 U/g TGase pre-crosslinked for 2–3 h) improved the emulsification and gel properties to varying degrees: the nanoparticle and emulsification performance increased by 24.35% and the storage modulus of the gel increased by 288%. Furthermore, the surface charge of SPI increased due to the crosslinking impact of TGase, indicating a considerable rise in the surface electrostatic potential. Simultaneously, the protein surface exhibited a substantial increase in hydrophobicity, while the level of free sulfhydryl groups reduced. These changes indicate that TGase enzymatic crosslinking could significantly improve the structural stability of nanoparticles by enhancing the generation efficiency of covalent bonds between protein molecules.

## 1. Introduction

Soy protein is a prevalent dietary protein source, with soy protein isolate (SPI) being a significant commercial soy protein product known for its diverse health benefits such as lowering cholesterol, regulating glucose homeostasis, and preventing disease [1]. The widespread usage of SPI in various food applications can be ascribed to its functional qualities, such as solubility, gelation, and emulsification. However, it suffers from poor stability and low solubility and bioavailability in the process of utilization. Therefore, it is necessary to process SPI into nanoparticles using appropriate methods to increase its specific surface area, improve surface modifiability, and endow the nanoparticles with excellent properties for use as carriers.

The two primary globular proteins in SPI are β-Accompanied Soya Globulin (7S) and Soya Globulin (11S). The most common techniques for creating nanoparticles from globular proteins include emulsion evaporation, antisolvent precipitation, thermal induction, and electro spraying [2]. Compared with traditional physical and chemical methods, protein nanoparticles prepared by enzyme crosslinking based on covalent bonds between proteins may be denser and more stable. Moreover, the crosslinking reaction conditions are mild and have the benefits of low energy consumption, a high nutrient retention rate, minimal chemical residue, high protein biocompatibility and safety, and a lack of impurities such as metal fragments that may be introduced by traditional physical methods. For example, compared with physical methods such as ultrasound, TGase crosslinking treatment is carried out under mild conditions in which the degree of protein crosslinking can be more accurately regulated, excessive degradation or aggregation problems can be avoided, and its nutritional composition and biocompatibility can be maintained [3]. However, research on enzyme-crosslinked nanoparticles has predominantly focused on animal-derived proteins, such as casein, whey, and ovalbumin, with few studies involving plant-based proteins [4,5].

Transglutaminase (TGase), a widely utilized protein crosslinking enzyme in the food industry, facilitates crosslinking between protein molecules without significantly altering their nutritional and functional properties, thus holding high application value in the food field [6]. The mechanism underlying TGase activity involves catalyzing the acyl transfer reaction between the γ-carboxamide group of glutamines (Gln) residues and the ε-amino group of lysine (Lys) residues within proteins. This reaction leads to the formation of ε-(γ-glutamyl) lysine and other covalent peptide bonds, facilitating both intermolecular and intramolecular crosslinking [7]. By creating intermolecular peptide connections between alcohol-soluble proteins, TGase-catalyzed crosslinking improves the mechanical characteristics of the interface, according to research by Kaczynska et al. [8]. In addition, Sun et al. [2] synthesized nanoparticles from soy protein via mTGase-induced crosslinking. Compared with SPI-stabilized emulsion, covalently bonded nanoparticles significantly improved the stability of the emulsion. These reports provide feasible evidence for studying the effect of TGase to improve the emulsification and gelation behavior of SPI nanoparticles. The impacts of TGase concentration and crosslinking time on SPI’s physical, chemical, and functional properties were the primary goals of the study. In this study, SPINPs were first crosslinked via heat treatment, and the proteins were then crosslinked using varying TGase concentrations and crosslinking durations. The particle size, zeta potential, surface hydrophobicity, and secondary structure of SPINPs with different crosslinks were determined and interpreted to further explore their effects on the emulsification properties and gelation behavior of SPINPs. This research elucidates the role and mechanism of TGase crosslinking in SPI, thereby offering a theoretical foundation for the creation and use of items made from soy.

## 2. Materials and Methods

### 2.1. Materials

Defatted soybean meals were obtained from Yu wang Company, located in Shandong, China. Transglutaminase (TGase), with an enzyme activity of 1000 U/g, was obtained from Beijing Solarbio Technology Co., Ltd. (Beijing, China). 8-anilinonaphthalene-1-sulfonic acid (ANS) and all other chemical reagents used were of analytical grade.

### 2.2. Methods

#### 2.2.1. Preparation of Soy Protein

After dispersing the defatted soybean meals (*w/v*) in phosphate buffer (10 mM) at a 1:10 ratio, 2 M NaOH was used to bring the pH to 8.0. To remove insoluble materials, the mixture was centrifuged for 25 min at 8000 rpm after being stirred for 4 h at room temperature. After adding 2 M HCl to bring the supernatant’s pH down to 4.5, it was allowed to stand for four hours before being centrifuged for twenty-five minutes at 8000 rpm. The sediment was collected, washed with water three times, dissolved (bringing the pH down to 7.0), and then lyophilized. The Kjeldahl technique yielded a protein content of 90.60% (dry basis) and a N factor of 6.25.

#### 2.2.2. TGase Crosslinking of Soy Protein

##### Heat Treatment of Soy Protein Nanoparticles

To achieve complete protein dissolution, SPI powder was equally distributed in phosphate buffer, agitated overnight at room temperature, centrifuged at 4000 rpm for 20 min at 4 °C, and filtered (Sartorius, Minisart, 0.45 μm) to eliminate any remaining proteins. After the Bradford Protein Assay Kit (Beijing Solarbio Science & Technology Co., Ltd., Beijing, China) was used to measure the protein solutions’ concentration, they were diluted to 10 mg/mL.

To stop water evaporation, 40 mL of the sample was heated in a sealed bottle at 95 °C for 30 min while being stirred magnetically. The sample was then promptly cooled to room temperature for further experimental procedures after the heat treatment.

##### Preparation of Soy Protein Nanoparticles with Different TGase Concentrations

The heat-treated SPI samples were mixed with TGase enzyme at concentrations of 2.5, 5, 7.5, 10, and 12.5 U/g of protein.

The crosslinking reaction was conducted at 50 °C for 2 h following the addition of TGase. Respectively, each aliquot was heated at 90 °C for 10 min to inactivate TGase.

##### Preparation of Soy Protein Nanoparticles with Different TGase Crosslinking Times

The SPI samples, which had undergone heat treatment, were mixed with TGase at a 5 U/g protein concentration. To assess the crosslinking activity of SPI under various treatment conditions, the crosslinking reaction was initiated following the addition of TGase at 50 °C for 1 h, 2 h, 3 h, 4 h, and 5 h. Following this, the samples underwent a deactivation procedure that involved heating them for 10 min at 90 °C.

### 2.3. Sodium Dodecyl Sulfate–Polyacrylamide Gel Electrophoresis (SDS-PAGE)

SDS-PAGE analysis was carried out using the methodology described in our previous study [9]. This technique utilizes a 5% (*w/v*) stacking gel and a 12% (*w/v*) separating gel, as reported in prior studies. The SPI samples with different TGase concentrations (Section 2.2) and TGase crosslinking periods were diluted to 10 mg/mL using deionized water, and then mixed with Tris HCl buffer. To guarantee total denaturation, the samples were exposed to boiling water for five minutes. After 5 μL of the protein samples were centrifuged to eliminate insoluble contaminants, they were put onto the gel. The separation gel and stacking gel were electrophoresed at 80 V and 110 V. The gel was then immersed in Coomassie brilliant blue staining solution and stained on an oscillator for 30 min. Lastly, the gel was dipped into the decolorizing solution and a shaking table was used to decolorize it until the gel’s strips became plainly visible.

### 2.4. Measurement of Particle Size and Zeta Potential

Using a Zetasizer Pro laser light scattering device (Malvern Instrument Ltd., Worcestershire, UK), DLS was used to evaluate the particle size and zeta potential.

The protein nanoparticle samples were adjusted to a concentration of 1 mg/mL using 10 mM phosphate buffer and subsequently filtered through a 0.45 μm water membrane (Sartorius, Minisart, Gottingen, Germany) to eliminate large particle impurities. The diluted sample was moved to a glass tube for measurement. Each sample was equilibrated for 60 s at 20 °C for an average of 15 measurements lasting for 10 s. Natural protein served as the control, and all measurements were conducted in triplicate.

### 2.5. Analysis of Fluorescence Spectra in Protein Samples

The spectral analysis was carried out following a previously described method with minor changes [10]. The protein sample was excited at 280 nm, and the emission spectrum was scanned from 300 nm to 450 nm to obtain fluorescence spectrum data. Three independent replicates were used in the experiment to guarantee the accuracy and repeatability of the findings.

### 2.6. Determination of Surface Hydrophobicity (H_0_)

The H_0_ of the soy protein nanoparticles was determined using the ANS fluorescent probe method. Refer to a previous study for the specific operation steps [11]. The protein nanoparticles were continuously diluted with 10 mM phosphate buffer to prepare a sample solution with a concentration range of 0.05~0.3 mg/mL.

Following the addition of 20 μL of an 8 mM ANS solution to the diluted protein samples (4 mL), the fluorescence intensity was subsequently measured using a fluorescence spectrophotometer at an excitation wavelength of 390 nm (λex) and an emission wavelength of 470 nm (λem). By computing the initial slope of the curve connecting fluorescence intensity and protein content, the H_0_ of the soy protein nanoparticles was ascertained.

### 2.7. Determination of Free Sulfhydryl (-SH) Group Content

Following the method of Huang et al. [12], the free sulfhydryl content (-SH) in the protein solution was determined, and the protein solution concentration was adjusted to 3 mg/mL using Tris Gly buffer, centrifuged at 5000× *g* for 15 min, immediately mixed with Ellman’s reagent (4 mg DTNB/mL phosphate buffer) in a 100:1 ratio, and stored in the dark for 15 min. The absorbance value was measured at 412 nm with an ultraviolet spectrophotometer (UV-1780, Shimadzu, Kyoto, Japan).

### 2.8. Fourier-Transform Infrared (FTIR) Spectroscopy

Following the reported method with modifications [4], 200 mg of KBr powder and 2 mg of the freeze-dried sample powder were combined thoroughly and formed into tablets. A Nicolet iS20 FTIR spectrometer (Thermo Fisher Scientific, Waltham, MA, USA) was used to record 32 scanning interferograms at a resolution of 4 cm^−1^, covering a wavenumber range from 4000 to 400 cm^−1^. Omnic 8.0 software (Thermo Fisher Scientific Inc., Madison, WI, USA) and Peakfit 4.12 (Systat software, San Jose, CA, USA) were used to analyze the data. The Omnic 8.0 software was used to transform the spectral coordinates, and Peakfit 4.12 was employed to process the spectral data, yielding the Fourier self-deconvolution and second derivative of the amide I band within the 1700–1600 cm^−1^ range.

### 2.9. Emulsifying Activity Index (EAI) and Emulsifying Stability Index (ESI)

The preparation of the emulsions was based on methods described elsewhere, using the nanoparticles prepared as described above [2]. Briefly, the 30 mL sample was adjusted to a uniform protein concentration of 10 mg/mL. To make the lotion, it was then completely combined with 10 mL soy oil using a homogenizer set to 18,000 rpm for two minutes. The sample’s emulsification performance was evaluated using the methodology put forth by Li et al. [13]. A mixture consisting of 50 μL lotion (sampled 0.5 cm from the bottom of the container) with 5 mL of 1 mg/mL SDS solution was prepared via eddy current. Using 1 mg/mL SDS as a blank control, the absorbance of the mixture was measured at 500 nm at both 0 and 20 min, and the EAI and ESI were calculated according to the following formula:

(1) $$EAI(m^{2}/g)=\frac{2\times 2.303\times A_{0}\times D}{\varphi \times C\times 10^{4}}$$

(2) $$ESI\left(\%\right)=\frac{A_{20}}{A_{0}}\times 100$$

where A_0_ and A_20_ represent the absorbance values at 500 nm for 0 min and 20 min, respectively. C is the concentration of the sample before emulsification; Φ is the volume fraction of oil in the emulsion, taken as 0.25 here; and D is the dilution multiple before measurement. Each sample was measured three times.

### 2.10. Rheological Examination of SPI Gels

A MARS60 HAAKE rheometer (Thermo Fisher Scientific, Waltham, MA, USA) with parallel plates (diameter = 35 mm, gap = 1 mm) was used to conduct the rheological experiment. The samples were quickly moved onto the rheometer’s parallel plate after being treated using the previously outlined method to a concentration of 60 mg/mL. Water evaporation was stopped by low-viscosity silicon oil, which was left to stand until the gel was ready for measurement.

### 2.11. Statistical Analysis

Every experiment was conducted three times, and the mean ± standard deviation was used to represent the results. Duncan’s test (*p* < 0.05) was used to assess statistical differences between samples using IBM SPSS software (version 21.0, IBM, Chicago, IL, USA).

## 3. Results and Discussion

### 3.1. SDS-PAGE

**Figure 1:**
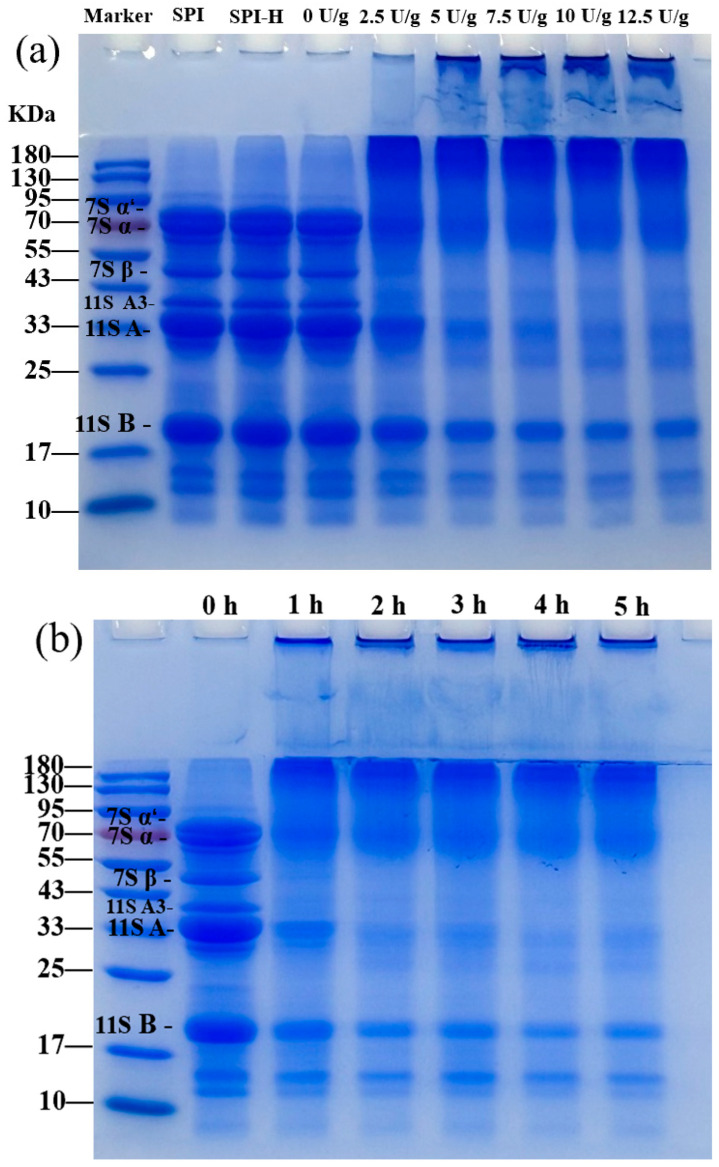
SDS-PAGE profile of SPI crosslinking products (SPI, SPI with heat treatment (**a**), SPI crosslinked with different TGase enzyme concentrations, SPI of TGase crosslinking at different crosslinking times (**b**). Marker: The left side displays the matching molecular weight.

The SDS-PAGE analysis (Figure 1) revealed the separation of SPI subunits before and after TGase crosslinking, with varying enzyme concentrations and crosslinking durations. The results indicated that SDS-PAGE effectively separated the 7S α′, 7S α, 7S β, 11S acidic, and 11S basic proteins in the soymilk samples [14]. The 33 kDa fraction contained 11S acidic protein and the 20 kDa fraction contained 11S basic protein. As can be seen in Figure 1a, for SPI without any treatment, the 7S (α′, α, β subunits) and 11S (acid and basic subunit) bands were significant, but no band was found at the top of the gel. The enzyme bands of TGase pretreated by heating exhibited little change, indicating that the presence of β-mercaptoethanol effectively disrupted most of the physical and chemical bonds, including disulfide bonds. It is worth noting that when SPI is crosslinked by TGase, 7S and 11S polymerize to synthesize higher-molecular-weight (>180 kDa) proteins that appear above the stacked gel (marked with black dashed lines in Figure 1). This is due to the ability of TGase, as an acyltransferase, to catalyze the acyl transfer reaction between Gln and Lys residues, promoting the intramolecular and intermolecular crosslinking of SPI and the formation of higher-molecular-weight polymers [3]. This finding is consistent with the observations of Chen et al. [15], who previously investigated the crosslinking reaction of whey protein concentrate treated with TGase and found that the concentrations of 7S and 11S proteins decreased as the amount of TGase and crosslinking time increased. The 11S A3 subunit disappeared first after crosslinking, followed by the gradual disappearance of the 7S α′, 7S α, and 7S β subunits. This sequence may be due to their spatial positions on the 11S molecular surface [16]. These findings suggest that 7S α′, 7S α and 7S β, and 11S A3 subunits are substrates of TGase. For native soy protein, due to its compact and hard spherical structure, only part of the 7S subunits is crosslinked with 11S acidic protein. The intensity of the 11S acidic protein band decreased significantly, whereas the intensity of the 11S basic protein band did not change significantly. This is due to the structural characteristics of the 11S globulin tetramer and the low glutamine/lysine content of 11S B subunits.

### 3.2. Zeta Potential and Particle Size of SPI

**Figure 2:**
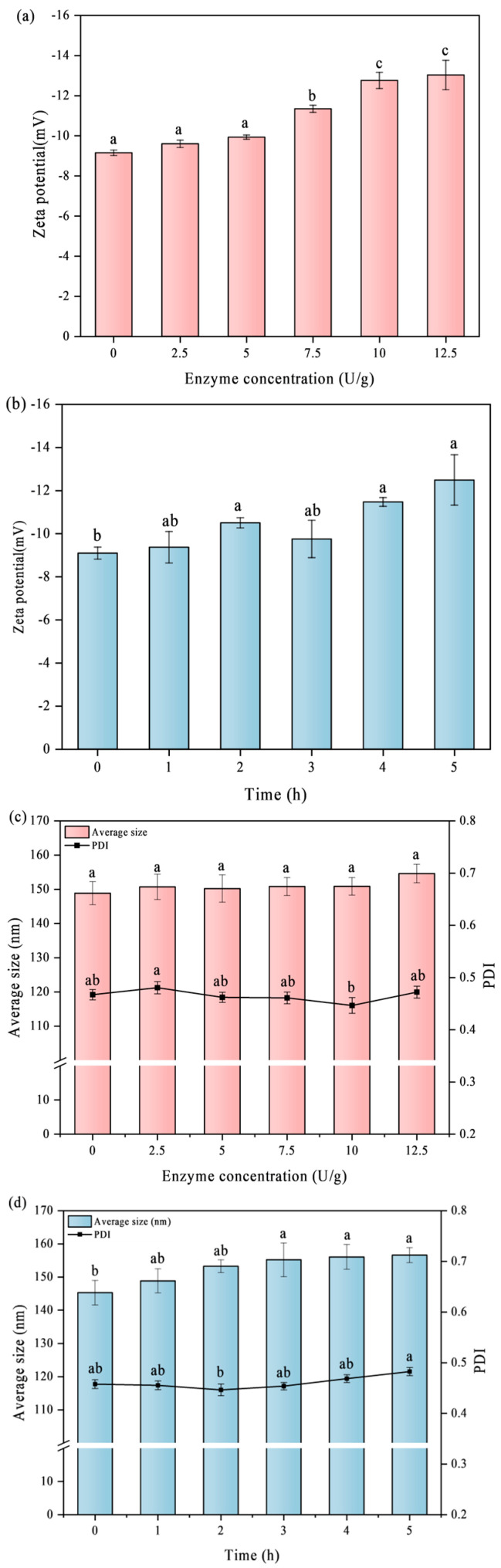
Effect of different TGase concentrations and crosslinking times on the size/PDI (**a**,**b**) and zeta potential (**c**,**d**) of SPI. Significant differences (*p* < 0.05) are indicated by different letters (a–c).

The surface potential is crucial for the formation and stability of protein emulsions [17]. Figure 2 shows the zeta potentials of the soy protein nanoparticles. It was observed that SPI was negatively charged, and as the enzyme treatment time and enzyme quantity rose, the absolute value of the potential increased noticeably from 9.10 ± 0.28 to 12.49 ± 1.17 and 13.03 ± 0.73, respectively. Additionally, the TGase-induced crosslinking was achieved by consuming positively charged lysine residues. The increase in the absolute value of zeta potential in the system indicates that the electrostatic repulsion is enhanced, which promotes the stability of the whole system [18]. This phenomenon aligns with previous findings, where Liu et al. [19] found that TGase crosslinking resulted in an increase in the absolute potential of faba bean protein nanoparticles. Figure 2c,d shows the particle size of SPINPS treated with enzyme. The size is primarily distributed between 145 and 160 nm, and the polydispersity index (PDI) is about 0.46, indicating a relatively uniform nanoparticle size.

### 3.3. Surface Hydrophobicity (H_0_)

**Figure 3:**
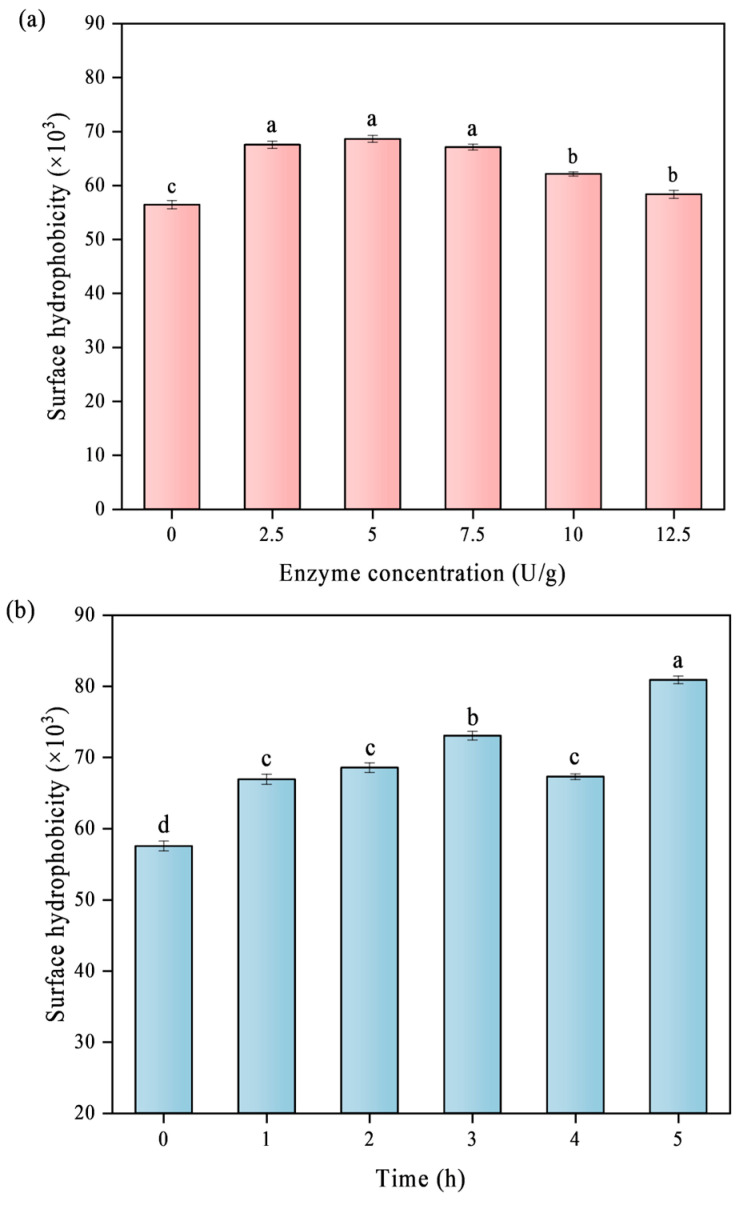
Effect of different TGase concentrations (**a**) and crosslinking times (**b**) on the H_0_ of SPI. Significant differences (*p* < 0.05) are indicated by different letters (a–d).

Protein H_0_ can reflect the degree of exposure of hydrophobic groups on its surface and directly determine the interaction ability between protein molecules, and is one of the key parameters to evaluate the degree of protein denaturation [20]. Figure 3 shows the H_0_ of various particle surfaces as determined using ANS fluorescence probes. The primary factors influencing the alteration of surface hydrophobicity during the TGase crosslinking process are the exposure and rearrangement of hydrophobic groups. The hydrophobic areas of protein molecules are progressively revealed as the degree of crosslinking rises, improving the protein’s capacity to interact with organic solvents. It makes sense that TGase crosslinking exposes the hydrophobic regions of the protein. Moreover, the enhanced hydrophobicity is likely related to the deamidation process that occurs during TGase crosslinking, which leads to the loss of amino groups and diminishes the hydrogen bonds that hold proteins and water together, thereby increasing the exposure of hydrophobic areas [21]. In addition, the surface hydrophobicity initially rose and subsequently declined with increasing TGase concentration. The protein molecular weight increased after further crosslinking by TGase, while the hydrophobic regions in the supramolecule were rearranged and tended to form a more stable spatial structure, which led to a subsequent decrease in hydrophobicity. Prior research on the crosslinking of protein produced from faba beans with TGase has noted a similar phenomenon [19].

### 3.4. Fluorescence Spectroscopy

**Figure 4:**
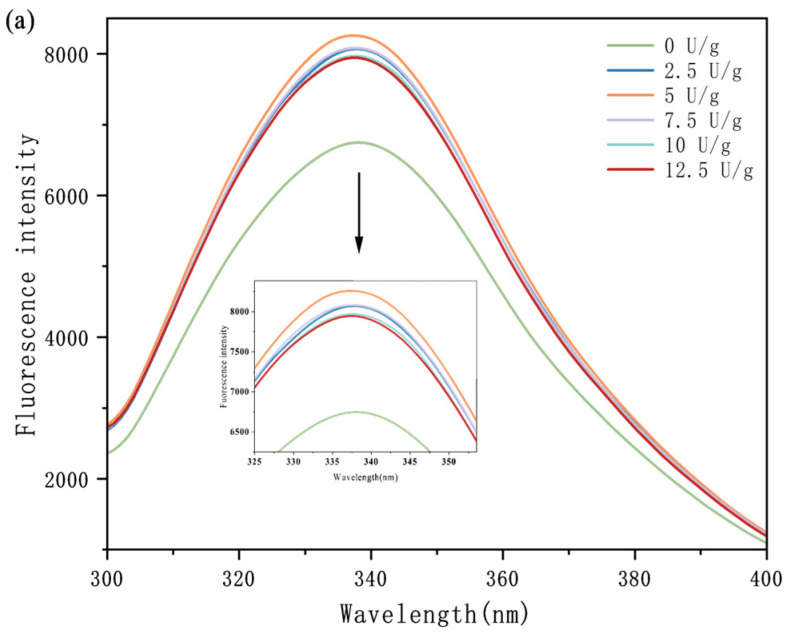

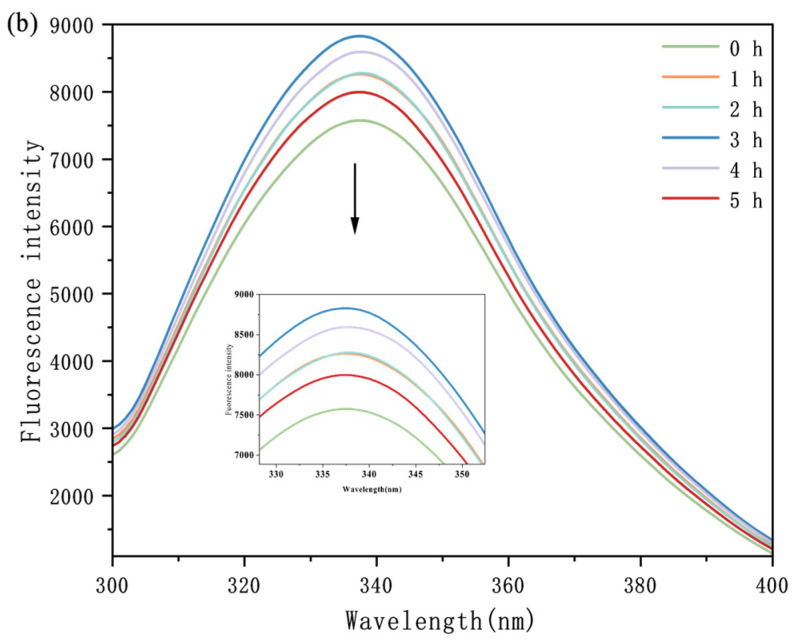
Endogenous fluorescence spectroscopy of SPI treated with different TGase concentrations (**a**) and crosslinking times (**b**).

Endogenous fluorescence spectroscopy is valuable for studying conformational shifts in proteins. Among the three fluorescent amino groups (Trp, Tyr, Phe) in proteins, Trp and Tyr residues are closely related to the conformational changes [22]. Therefore, endogenous fluorescence spectroscopy can be used to characterize changes in protein tertiary structure. The fluorescence spectra of samples (SPI treated with TGase crosslinking at different enzyme concentrations and crosslinking times) are shown in Figure 4. The fluorescence spectrum of SPI showed no significant red shift or blue shift, but the fluorescence intensity increased significantly (*p* < 0.05). The change in fluorescence quantum yield affected the fluorescence intensity, and the fluorescence quantum yield itself was regulated by protein structure. Enzyme activity exhibited a trend from rising to falling with an increase in enzyme content and an extension of crosslinking time. The increase stage of the fluorescence quenching period may be due to the catalysis of TGase, and tryptophan residues are buried in the protein, thus reducing its contact with water molecules and reducing the fluorescence quenching rate. With the continuous increase in the enzyme dosage and time, the observed decrease in fluorescence intensity may be attributed to the unfolding of the protein structure, which exposes more hydrophobic groups and tryptophan residues [23]. Hydrophobic interactions mainly affect the folding path and final confirmation of proteins and affect the stability of proteins by regulating the aggregation of non-polar residues, thus affecting the fluorescence characteristics. In addition, structural changes may increase the electrostatic repulsion force inside the protein, thereby reducing the fluorescence intensity, as suggested by Babiker [24]. Electrostatic interaction mainly affects the charge distribution between amino acid residues, and then affects the folding and stability of proteins. For example, the changes in Lys and Gln residues reflect the changes in charge distribution in proteins, which may be related to the covalent bonds formed in the TGase crosslinking process. In general, the fluorescence intensity of each crosslinking protein changed with the change in crosslinking conditions. The tertiary structure of the protein was significantly impacted by the crosslinking duration and enzyme dose. The results of synchronous fluorescence spectrum analysis reflected the effect of preheating on the amino acid microenvironment of soy protein. The change in SPI’s molecular structure leads to the change in the arrangement of hydrophobic entities on its surface [25].

### 3.5. Secondary Structure

**Figure 5:**
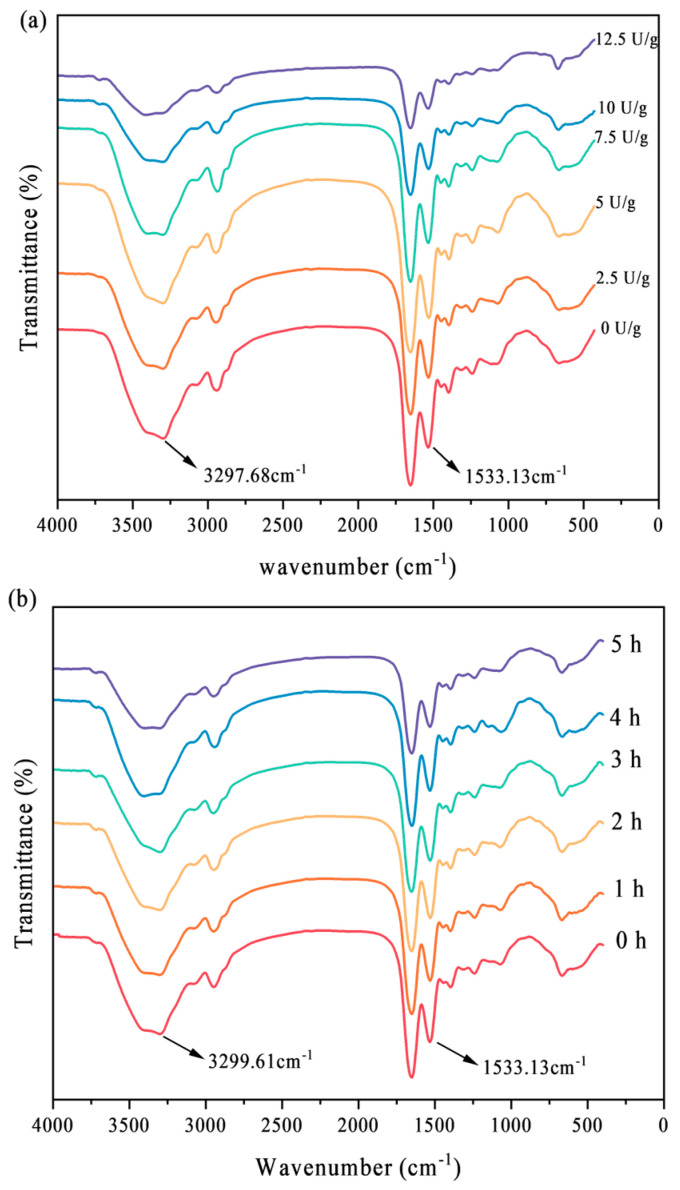
Infrared spectral analysis of SPI treated with different TGase concentrations (**a**) and crosslinking times (**b**).

The FTIR spectrum is depicted in Figure 5. The absorption peak at 3297 cm^−1^ is attributed to the stretching vibration of amido N–H and hydroxyl group–OH. With the extension of the enzyme crosslinking time, the highest specificity peak redshifted from 3299 cm^−1^ to 3405 cm^−1^; with the increase in enzyme concentration, the N-H specificity peak redshifted from 3297 cm^−1^ to 3420 cm^−1^. The N–H vibration in amido-NH_2_ is responsible for the absorption peak at 1533 cm^−1^ [26]. With the extension of the enzyme crosslinking time and the increase in enzyme concentration, these highest specific peaks redshifted from 1533 cm^−1^ to 1540 cm^−1^ and 1541 cm^−1^. This results from the acyl transfer process that forms iso peptide bonds between Gln and Lys residues [27], which aligns with the crosslinking mechanism between proteins and previous research reports [28].

The protein secondary structure was examined using FTIR spectroscopy. Table 1 displays the relative content of SPI’s secondary structure. The C–O stretching vibration and, to a lesser degree, the C–N stretching vibration were attributed to the amide I at 1600–1700 cm^−1^ [26]. The bands between 1660 and 1650 cm^−1^ are part of the α-helical structures’ distinctive absorption band. While 1640–1610 cm^−1^ and 1690–1670 cm^−1^ were the characteristics of β-sheet, 1700–1690 cm^−1^ and 1670–1660 cm^−1^ were the characteristics of β-turn, and 1650–1640 cm^−1^ were ascribed to random coil [29]. Overall, it is evident that the β-sheet structure increased with increasing enzyme content and the β-turn structure decreased after TGase crosslinking, which is in accordance with the findings of prior studies [30]. The β-sheet structure is crucial for maintaining the protein gel network, which provides theoretical support for the improvement of SPI gelation after TGase crosslinking. According to published research, proteins with more β-sheets will have longer sequences or more stable structures [31]. Meanwhile, as the crosslinking time increased, the β-sheet rose as a whole and showed more β-sheet structures at 3 h. This time-dependent increase in β-sheet content suggests that extended crosslinking promotes the formation of more stable and ordered protein structures. The increase in β-fold structure is closely related to the stability of the protein gel network, because β-fold can enhance the rigidity and stability of the protein matrix by forming extensive intermolecular hydrogen bonds [32]. This structural change directly led to an improvement in the rheological properties of SPI gel, indicating that TGase crosslinking significantly enhances the gelation performance of SPI by inducing the formation of β-folding structures.

### 3.6. Content of Free-SH

**Figure 6:**
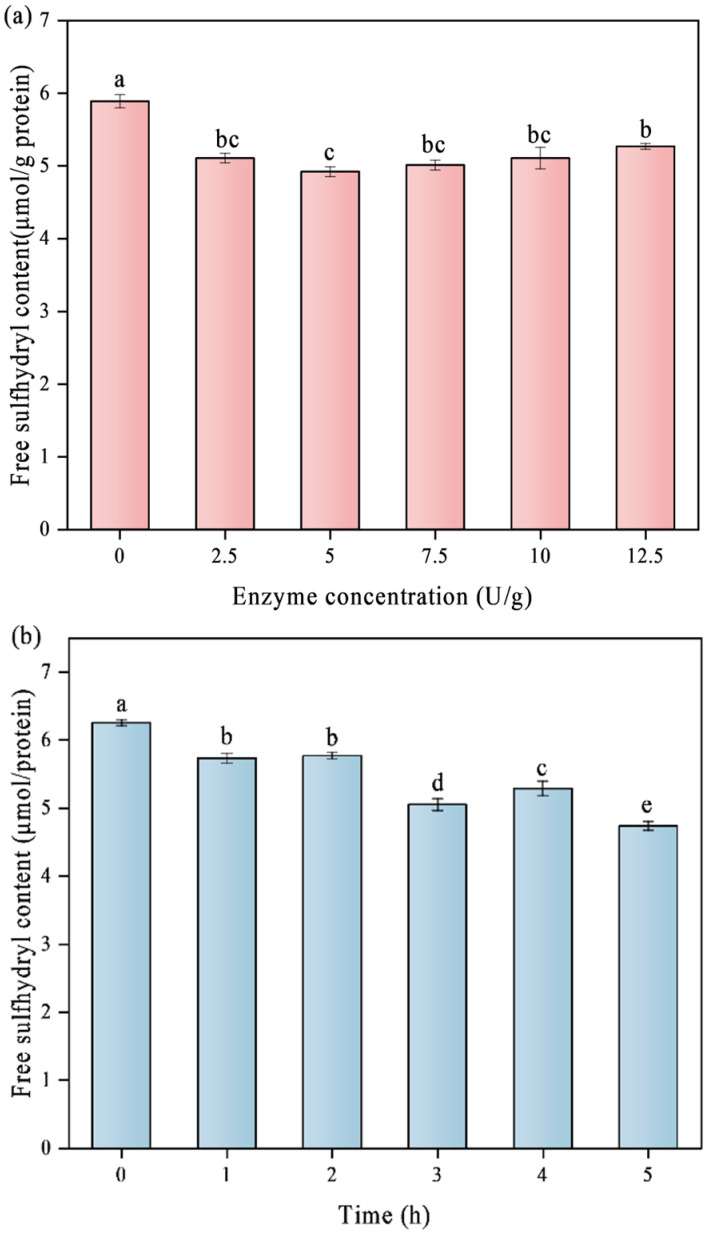
Effect of different TGase concentrations (**a**) and crosslinking times (**b**) on free sulfhydryl groups (S_0_) of SPI. Significant differences (*p* < 0.05) are indicated by different letters (a–e).

Free sulfhydryl groups (-SH) are predominantly found on the outside of protein molecules and are essential for their functional properties. When soy proteins are heated, subjected to high pH levels, or denaturated by enzymes, the number of these groups reduces [33]. Figure 6 shows the exposed SH of SPI at different time points and enzyme concentrations. The amount of -SH in the samples gradually decreased as the duration of TGase crosslinking and the concentration continued to increase. This might be explained by the crosslinking that TGase catalyzed, which promoted the formation of protein polymers. This process may lead to the breakage of disulfide bonds, with the exposed SH groups becoming embedded within the polymer structure [34]. The SDS-PAGE results in Figure 1 show the same changes. In addition, TGase crosslinking promotes the creation of disulfide bonds via oxidation by drawing sulfur-containing amino acids in the newly created crosslinked peptide chains closer together, lowering the amount of free thiol (-SH) present [35]. This result is in line with the findings of Li et al. [36], which showed that the SH content on the surface of proteins in soymilk decreased gradually with the addition of TGase during the preparation of tofu gels.

### 3.7. Emulsifying Properties

**Figure 7:**
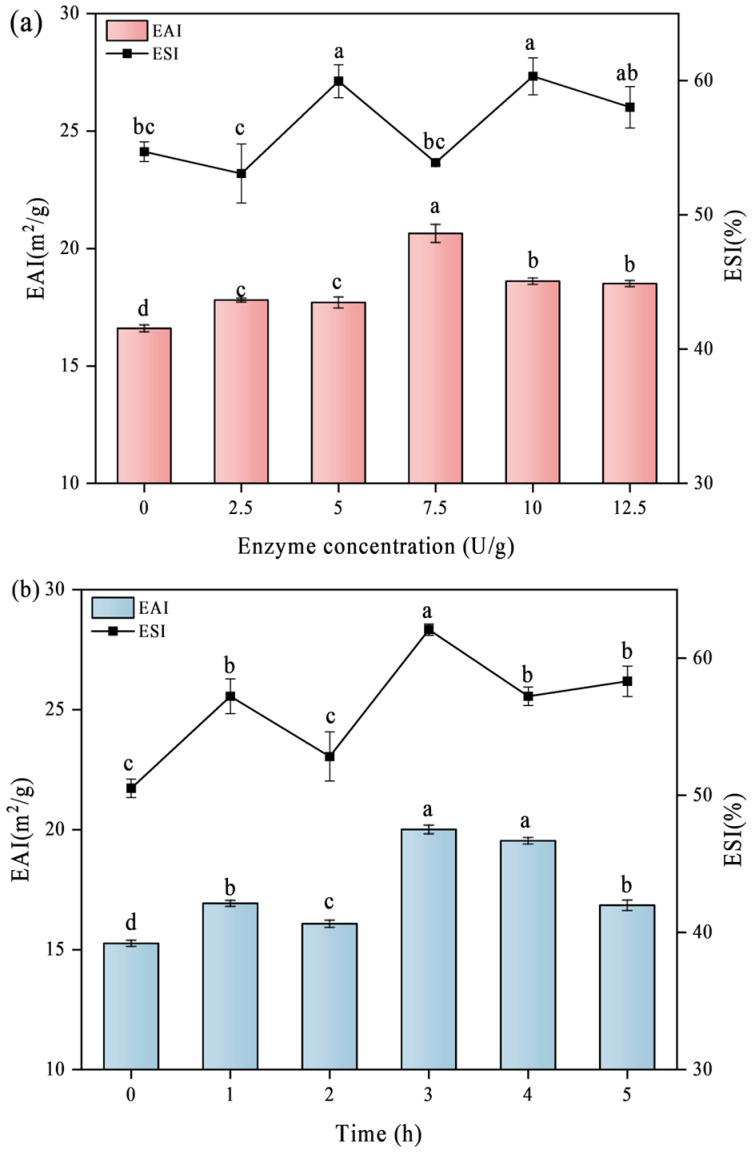
Effect of different TGase concentrations (**a**) and crosslinking times (**b**) on the EAI and ESI of SPI. Significant differences (*p* < 0.05) are indicated by different letters (a–d).

The EAI and ESI, representing the emulsification activity index and the emulsion stability index, respectively, are commonly used to assess the emulsification properties of emulsions. These indices are affected by various factors such as molecular weight, conformational stability, surface hydrophobicity, surface charge, and physical conditions like temperature, pH, and ionic strength [37]. The EAI can reflect the migration rate of protein particles to the emulsion interface. The EAI of crosslinked soy proteins increased, as shown in Figure 7a. The EAI increased by about 24.35% and 31.12% at an enzyme activity of 7.5 U/g (20.64 m^2^/g) and at a crosslinking time of up to 3 h (20.00 m^2^/g), respectively, and then showed a stable trend. This result is in line with Ali’s previous report that the TG enzyme improved the emulsifying activity of soy proteins [38]. At the same time, some studies reported that TG crosslinking improved the emulsifying properties of whey, seed, and soy proteins [39,40]. The TG-catalyzed crosslinking reaction increases the molecular weight of the proteins and promotes the exposure of hydrophobic groups, thus enhancing their surfactant properties. Larger aggregates of polymerized proteins provide a larger adsorption surface area at the oil–water interface. This enhances the EAI by increasing the electrostatic repulsion between droplets, thereby preventing agglomeration [41]. The decline in emulsification activity, attributed to high enzyme content or extended crosslinking time, may result from increased electrostatic repulsion due to the hydrolysis of glutamine and asparagine amide groups.

The ESI is closely associated with the hydrophobic–hydrophilic balance of protein molecules on the surfaces [42]. In this study, by improving the electrospray ionization strength of crosslinked soy protein, low flocculation, oligomerization, small droplet size, and high interface protein adsorption were achieved, which promoted the improvement of emulsion stability. The same findings were shown in the study of other legume proteins [2]. As shown in Figure 7b, the ESI can reach 60.40% at 10 U/g of enzyme content and 62.10% at 3 h, indicating the improved ability of stabilizing the emulsion without flocculation at the oil–water interface. However, with the increase of enzyme dosage and crosslinking time, the electrostatic repulsion between protein molecules was enhanced, which might affect the aggregation and arrangement of protein molecules at the oil–water interface, thus affecting the stability of emulsion [43].

### 3.8. Effects of Protein Structure Changes on the Gelation of SPI Crosslinked by TGase

**Figure 8:**
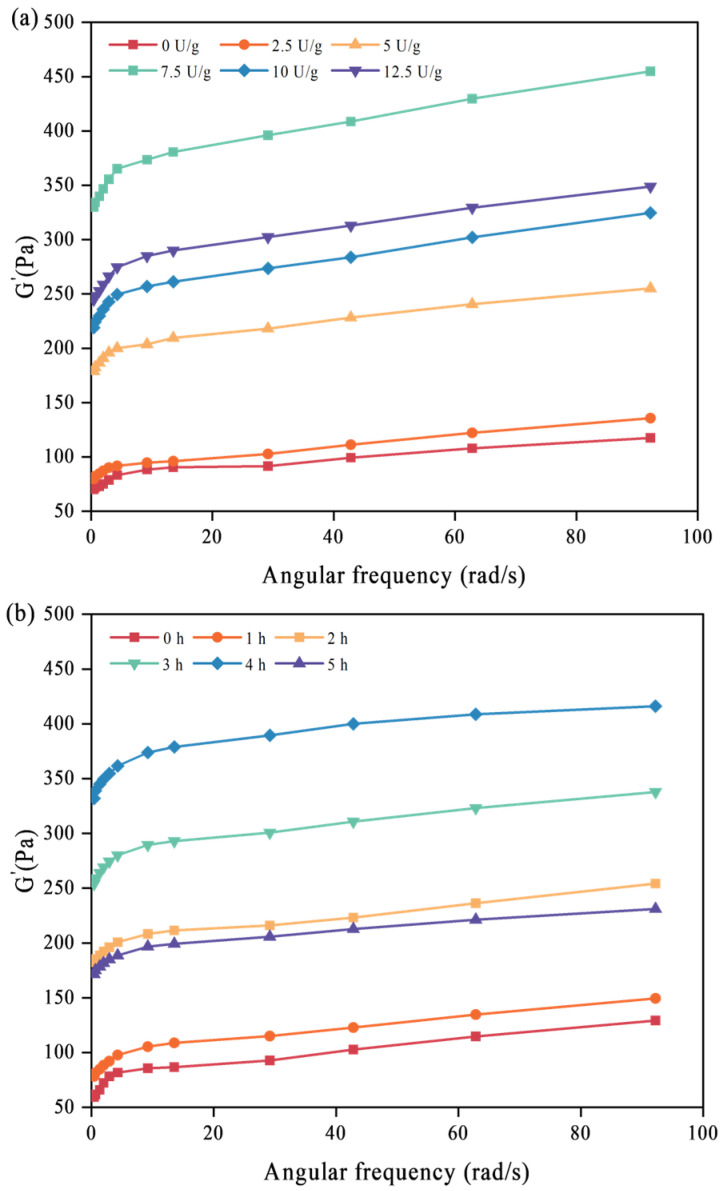
Effect of different TGase concentrations (**a**) and crosslinking times (**b**) on the storage modulus (G’) of SPI gels.

The effects of different crosslinking conditions on TGase-induced SPI gelation were studied using the rheological method. The relationship between storage modulus (G’) and angular frequency is given in Figure 8. The characteristic viscoelastic (solid-like) properties of all SPI gels were G′ > G″. In the whole angular frequency range of 1~100 rad/s, the value of G’ increases weakly with the increase in angular frequency ω, indicating that G’ is a covalent gel composed of “chemical” crosslinking, which is independent of frequency [44].

As the concentration of TGase increased and the pre-crosslinking time extended, the dependence of G’ on the angular frequency gradually weakened, indicating that TGase induced the formation of more lysine and glutamine in SPI, and chemical covalent bonds were formed in the SPI gel network. At this time, more chemical gels were involved in the protein gel structure, and the gel structure became more complex. The G’ value of the gel was maximum at a TGase concentration of 7.5 U/g or at a TGase crosslinking time of 4 h, which is similar to Spotti’s results [45]. Increasing the amount of enzyme or prolonging the crosslinking time led to excessive crosslinking of TGase, producing large aggregates, which might limit the rearrangement of protein molecules and other intermolecular interactions (e.g., hydrophobic interactions). The network structure of the gel may be damaged, with an inhomogeneous structure and reduced elastic properties of the gel. Therefore, an optimal degree of TGase pre-crosslinking can enhance the elastic modulus and strength of SPI gels. However, excessive crosslinking diminishes the strength of SPI gels.

## 4. Conclusions

In this study, TGase was used as a catalyst to create SPI nanoparticles via a crosslinking process when the soy protein isolate was heated to melting point. We examined the impact of TGase concentration and crosslinking time on the structural changes, emulsification, and gelation properties of SPI. The following main conclusions were drawn.

TGase accelerated the crosslinking between Lys and Gln residues of SPI, resulting in significant changes in SPI’s structure. These alterations include the increased intensities of endogenous fluorescence spectroscopy, heightened surface hydrophobicity, increased β-sheet structure and degraded β-turn, and decreased free sulfhydryl content. These results suggest that the moderate pre-crosslinking of TGase (e.g., 5–7.5 U/g TGase pre-crosslinking for 2 h or 5 U/g TGase pre-crosslinking for 2–3 h) might affect its emulsification and gel properties. The emulsifying activity (EAI) of nanoparticles increased by 24.35% and the G’ of the gel increased by 288%, mainly being attributed to the enhanced hydrogen bonding and hydrophobic interactions between protein molecules through TGase mediated crosslinking. Interestingly, excessive crosslinking or concentration of the enzyme led to oversized aggregates of the obtained SPI nanoparticles, weakening their adsorption in the gel network and discouraging the formation of a homogeneous gel network.

The study findings validated the hypothesis that appropriate TGase treatment can selectively modify the structure of SPI nanoparticles and optimize their functions (emulsion stability and gelation function), providing new possibilities for the application of soy protein in emulsion-based foods (such as beverages and sauces) and high-quality gel products (such as tofu and meat imitations). Future research can further explore the modification effect of TGase treatment on other plant proteins, the improvement of application performance in composite systems, and the long-term study of the stability of this modified SPI in actual complex food matrices.

## Figures and Tables

**Table 1:** Secondary structure in amide I region (1600–1700 cm^−1^) according to FTIR analysis.

	Secondary Structure Composition (%)			
Protein Sample	A-Helix (%)	Β-Sheet (%)	Β-Turn (%)	Random Coil (%)
TG-0 U/g	16.58 ± 0.27 ^a^	39.79 ± 0.33 ^c^	26.65 ± 0.11 ^a^	16.99 ± 0.05 ^b^
TG-2.5 U/g	16.43 ± 0.17 ^a^	40.39 ± 0.21 ^c^	25.47 ± 0.10 ^b^	17.72 ± 0.16 ^a^
TG-5 U/g	16.12 ± 0.28 ^a^	40.15 ± 0.27 ^c^	26.68 ± 0.13 ^a^	17.06 ± 0.15 ^b^
TG-7.5 U/g	16.34 ± 0.25 ^a^	41.50 ± 0.37 ^b^	25.52 ± 0.42 ^b^	16.64 ± 0.16 ^c^
TG-10 U/g	16.43 ± 0.23 ^a^	41.75 ± 0.26 ^b^	24.65 ± 0.11 ^c^	17.18 ± 0.14 ^b^
TG-12.5 U/g	16.08 ± 0.37 ^a^	42.96 ± 0.33 ^a^	24.57 ± 0.17 ^c^	16.40 ± 0.13 ^c^
TG-0 h	16.35 ± 0.20 ^b^	37.90 ± 0.25 ^b^	27.92 ± 0.27 ^a^	17.84 ± 0.31 ^c^
TG-1 h	18.17 ± 0.23 ^a^	38.45 ± 0.37 ^b^	24.65 ± 0.16 ^d^	18.74 ± 0.57 ^b^
TG-2 h	17.76 ± 0.38 ^a^	38.35 ± 0.30 ^b^	26.05 ± 0.11 ^c^	17.85 ± 0.04 ^c^
TG-3 h	16.96 ± 0.24 ^b^	39.67 ± 0.31 ^a^	24.82 ± 0.21 ^d^	18.50 ± 0.07 ^b^
TG-4 h	16.72 ± 0.30 ^b^	38.24 ± 0.26 ^b^	25.70 ± 0.38 ^c^	19.35 ± 0.35 ^a^
TG-5 h	15.69 ± 0.23 ^c^	38.59 ± 0.26 ^b^	27.11 ± 0.20 ^b^	18.61 ± 0.28 ^b^

Significant differences (*p* < 0.05) are indicated by different letters (a–d).

## Data Availability

The original contributions presented in this study are included in the article. Further inquiries can be directed to the corresponding author.
